# A Systematic Review on Prosthodontic Rehabilitation of Hemimandibulectomy Defects

**DOI:** 10.7759/cureus.44647

**Published:** 2023-09-04

**Authors:** Surender Kumar, Hemlata Dwivedi, Bishnupati Singh, Awanindra K Jha, Reeta Jain, Sunil Singh

**Affiliations:** 1 Department of Prosthodontics, Dental Institute Rajendra Institute of Medical Sciences, Ranchi, IND; 2 Department of Prosthodontics, Rama Dental College and Hospital, Kanpur, IND; 3 Department of Orthodontics and Dentofacial Orthopedics, Dental Institute Rajendra Institute of Medical Sciences, Ranchi, IND; 4 Department of Prosthodontics, Genesis Institute of Dental Sciences and Research, Ferozepur, IND; 5 Department of Prosthodontics, Dental College Azamgarh, Azamgarh, IND

**Keywords:** maxillofacial prosthodontics, cantor curtis classification, prosthetic/prosthodontic rehabilitation, mandibular reconstruction, hemimandibulectomy

## Abstract

Restoration of hemimandibulectomy defects following tumour extirpation to restore oral function is a herculean task for practitioners. Prosthetic treatment alternatives available for rehabilitation of acquired hemimandibulectomy defects according to mandibular reconstruction type and extent (Cantor-Curtis classification) are unclear. This systematic review aims to assess the spectrum of prosthodontic rehabilitation approaches with regard to reconstruction type and extent of mandibular surgical defects. The databases incorporated for literature search were Google Scholar and Medline (PubMed). Relevant search terms for hemimandibulectomy and reconstruction with prosthetic rehabilitation were used. Two reviewers independently assessed the articles using eligibility criteria; published case reports and case series in the English language and depicting prosthodontic treatment modality of patients greater than 15 years were included. A total of 202 records were identified from the database search of which 19 duplicates were removed. The remaining articles were assessed for eligibility, and 55 articles (comprising 58 cases) were finally included in the study. This review revealed various prosthetic alternatives ranging from guide flange, twin occlusion, palatal ramp, conventional to hybrid partial and complete dentures to implant-supported prosthesis including a few innovative prosthetic approaches. This systematic review provides a plethora of prosthodontic rehabilitation approaches according to the extent of hemimandibular surgical defect and type of reconstruction. This will facilitate practitioners and prosthodontists in sequential treatment planning and management of hemimandibulectomy cases in their routine practice.

## Introduction and background

Surgical excision of various malignant or benign tumours like ameloblastoma, osteosarcoma and various injuries of the maxilla and/or mandible has resulted in partial or total maxillofacial defects involving hard and soft tissues. These conditions deteriorate oral function, aesthetics and comfort leading to impaired quality of life. Due to extensive surgical resection, the surface area required for adequate retention of the prosthesis is remarkably reduced. The radiotherapy adjunct with surgery further deteriorates the load-bearing capacity of the underlying denture-supporting tissue, and thus, the prosthodontic rehabilitation of these patients becomes an uphill task. Various classifications of hemimandibulectomy defects based on the nature and extent of mandible resection are available, but the Cantor and Curtis (CC) classification devised in the 1970s was widely observed in the majority of the articles studied. This system classifies defects based on remaining structures into six classes [1].

Restoration of the hemimandibular defect is a multidisciplinary approach involving an onco-surgeon, oral maxillofacial surgeon, prosthodontist, speech therapist, physiotherapist, etc. Treatment varies according to the type of mandibular reconstruction (hard tissue graft; fibula, iliac, etc. or soft tissue graft; pectoralis major myocutaneous flaps, etc.). Various prosthetic options are available including implant-supported prosthesis, endoprosthesis, guiding flange, twin occlusion prosthesis, etc. to improve a patient’s mastication, speech, aesthetics and lifestyle [2]. Despite these treatment recommendations, the ideal prosthetic rehabilitation of patients diagnosed with hemimandibulectomy is challenging and indecisive among practitioners. A review of the literature regarding functional outcomes related to prosthetic treatment after hemimandibulectomy has been performed previously, but a published systematic review on this topic is lacking. The present systematic review was a comprehensive review of prosthetic treatment approaches in hemimandibulectomy patients, and the purpose of the systematic review was to give treatment recommendations according to the type of mandibular reconstruction and the extent of the defect on the available evidence.

## Review

Methodology

Review Protocol

The systematic literature search was performed according to the Preferred Reporting Items for Systematic Reviews and Meta-analysis (PRISMA) guidelines [3] and was registered at the International Prospective Register of Systematic Reviews (PROSPERO-CRD42021264928).

Literature Search Strategy

An initial search was conducted on June 30, 2021, by using electronic databases of Medline (PubMed) and Google Scholar by two independent researchers (SK and SS) for published articles from January 1, 2010, to June 30, 2021, as per inclusion criteria. The database Medline (PubMed) was searched with the following keywords for Medical Subject Headings (MeSH) terms: "hemimandibulectomy", "rehabilitation", "prosthetic" and combinations of these keywords were used for Google Scholar with appropriate filters (Table 1).

**Table 1:** Systematic search strategy MEDLINE: Medical Literature Analysis and Retrieval System Online; MeSH: Medical Subject Headings.

MEDLINE (PubMed) Search
((hemimandibulectomy OR mandible resection OR segmental mandible resection OR partial mandibulectomy OR marginal mandibular osteotomy OR ("Mandibular Osteotomy/rehabilitation"[Mesh] OR "Mandibular Osteotomy/therapy"[Mesh])) AND (rehabilitation OR Reconstruction OR reestablishment OR management OR intervention OR("Rehabilitation/methods"[Majr:NoExp] OR "Rehabilitation/rehabilitation"[Majr:NoExp] OR "Rehabilitation/therapy"[Majr:NoExp]))) AND (prosthetic OR prosthodontic OR prosthesis OR dental prosthesis OR dental care OR ("Mandibular Prosthesis/methods"[Mesh] OR "Mandibular Prosthesis/rehabilitation"[Mesh] OR "Mandibular Prosthesis/therapy"[Mesh])) AND ((ffrft[Filter]) AND (casereports[Filter]) AND (2010/1/1:2021/6/30[pdat]) AND (english[Filter])) NOT Review Filters: Free full text, Case Reports, English, from January 1, 2010, to June 30, 2021

Screening and Selection

All published case reports and case series on human subjects having hemimandibulectomy defects, fulfilling the inclusion criteria and depicting a type of prosthetic rehabilitation were considered. Only full-text articles published in the English language were included. Original research, clinical trials, laboratory studies, animal studies, editorials, questionnaire studies and reviews were excluded. Titles and abstracts were screened (HD and SK) according to the inclusion criteria, and those with unclear methodology were included in the full-text assessment (Table 2).

**Table 2:** Inclusion and exclusion criteria TMJ: temporomandibular joint.

Inclusion Criteria	Exclusion Criteria
Patient above 15 years of age	Patient 15 years or less
Hemimandibulectomy following surgical resection	Total mandibulectomy
Hemimandibulectomy of both edentulous and partially edentulous arches Cantor and Curtis classification	TMJ ankylosis

Risk of Bias Assessment

Quality assessment of all the relevant studies included in the present review was performed by two reviewers (HD and BS) according to the Joanna Briggs Institute (JBI, Adelaide, Australia) [4]. This JBI critical appraisal tool comprises eight questions for case reports and 10 questions for case series that assess specific domains to determine the potential risk of bias and could be answered with ‘yes’, ‘no’ or ‘unclear’(Supplementary Appendix 1). Reports scoring less than four questions out of eight as ‘yes’ (<50% JBI) in case reports and less than five answers as ‘yes’ out of 10 questions in case series were denoted as high risk of bias and were excluded. Any disagreements between reviewers were discussed and resolved by consensus. If no consensus could be reached, a third reviewer (RJ) gave a binding verdict. The risk of bias in individual studies was determined with the following cut-offs: low risk of bias if 70% of answers scored yes, moderate risk if 50% to 69% of questions scored yes and high risk of bias if yes scores were below 50% and were excluded [5].

Data Extraction

The relevant studies obtained following screening were categorised into two groups: case reports and case series. Two reviewers (HD and SK) accomplished data extraction individually, while AJ checked the data: author name, year of publication, JBI score, age, gender, extent of defect (CC classification), name and type of prosthesis in both arches, reconstruction type (if any), surgical scarring, radiotherapy, follow-up period and adverse effect.

Results

Study Characteristics

**Figure 1:**
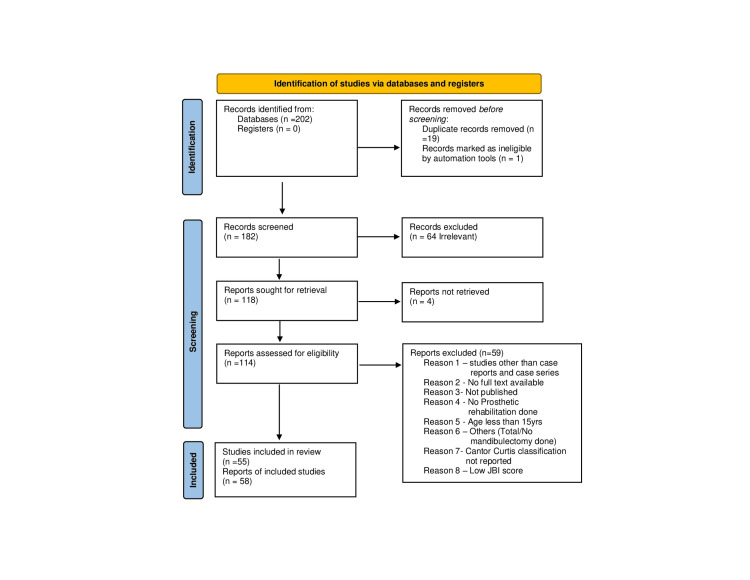
PRISMA flow diagram depicting the literature selection process PRISMA: Preferred Reporting Items for Systematic Review and Meta-analysis; JBI: Joanna Briggs Institute.

The initial literature search from the selected databases revealed 202 records from which 19 duplicates were identified and removed. After the screening of titles and abstracts, 55 articles (58 cases) with moderate to low risk of bias were finally included after quality assessment (Figure 1).

A total of 58 individuals (41 males and 17 females) with hemimandibular defect according to the Cantor-Curtis classification (class I: n= 3; class II: n= 25; class III: n= 24; class IV: n=6; class V and VI: n=0) were finally included. Pathak et al. [6] described a case of resection of the lateral portion of the mandible without subsequent augmentation as class VI; although according to CC classification, it fits in class II; therefore, in the present review, it was considered in class II defect. Out of 58 cases, 18 mentioned surgical scarring and 25 reported radiotherapy (Tables 3, 4) [6-60].

**Table 3:** Summary of the data collected from case reports NA: not applicable; NR: not reported; R: removable; F: fixed; PMMC: pectoralis major myocutaneous; CCC: Cantor-Curtis classification; JBI: Joanna Briggs Institute; MMF: maxillomandibular fixation; Ti: titanium; FG: fibula graft; IG: iliac graft; RG: ramus graft; MRP: maxillary ramp prosthesis; ISP: implant-supported prosthesis; MGFP: mandibular guide flange prosthesis; CD: complete denture; RPD: removable partial denture; FPD: fixed partial denture; OVD: overdenture; ARC: acetal resin clasp; RT: radiotherapy; CPD: cast partial denture.

Authors		JBI (%)	Age	Sex	CCC	Maxilla		Mandible		Mandibular Reconstruction	Surgical Scarring	RT	Recall Visit	Adverse Effect
						Name of Prosthesis	Type of Prosthesis	Name of Prosthesis	Type of Prosthesis					
Pathak et al. [6] (2015)		62.5	39	Male	II	NR	NR	MGFP	R	NR	NR	Yes	NR	NR
Gaur et al. [7] (2020)		100	68	Male	I	ISP with twin occlusion	F	ISP FPD	F	Yes (radial forearm flap)	YES	Yes	3 years	None
Kumthekar et al. [8] (2020)		100	75	Male	II	ISP OVD with MRP	R	ISP RPD	R	NR	NR	Yes	12 months	NR
Gupta et al. [9] (2016)		100	72	Male	II	CD with MRP	R	ISP OVD	R	Not done	NR	Yes	6 months	Yes and corrected
Patel et al. [10] (2020)		100	68	Male	II	FPD	F	ISP OVD	R	No	NR	NR	3 month	NR
Hindocha and Dudani [11] (2017)	100		72	Male	II	CD with twin occlusion	R	Mandibular segmental denture	R	Not done	NR	Yes	15 days	NR
Coutinho et al. [12] (2020)	100		74	Male	III	CD with twin occlusion	R	CD	R	Not done	Yes	Yes	3 months	Yes and corrected
Sampat et al. [13] (2020)	87.5		75	Male	III	CD with twin occlusion	R	CD	R	Not done	Yes	Yes	1 week	Yes and corrected
Arora et al. [14] (2013)	100		55	Male	II	RPD with twin occlusion	R	RPD	R	Yes (PMMC)	Yes	NR	Yes (duration NR)	None
Shashidhara et al. [15] (2015)	100		50	Female	II	CD with twin occlusion	R	CD	R	Yes (PMMC)	NR	Yes	3 months	None
Talukder et al. [16] (2017)	75		31	Male	III	Twin occlusion	R	NR	NR	NR	Yes	NR	NR	NR
Koralakunte et al. [17] (2015)	87.5		55	Female	II	MRP with twin occlusion	R	RPD with bobby pins	R	Yes (PMMC)	Yes	NR	6 months	NR
Sureja et al. [18] (2014)	87.5		47	Male	III	Twin occlusion	R	NR	NR	NR	Yes	NR	NR	NR
Sahu et al. [19] (2017)	87.5		56	Male	II	CD with twin occlusion	R	CD	R	Yes (PMMC)	NR	NR	6 months	Yes and corrected
Singh and Kumari [20] (2019)	87.5		58	Female	II	Twin occlusion	R	MGFP	NR	Yes (myocutaneous flap)	Yes	NR	6 months	Yes and corrected
Marathe and Kshirsagar [21] (2016)	87.5		44	Female	II	CPD with twin occlusion	R	MGFP followed by CPD with attachment	R	Not done	Yes	Yes	4 years	NR
Atodaria et al. [22] (2014)	100		46	Male	III	Twin occlusion prosthesis	R	CD	R	Yes (soft tissue)	NR	Yes	Yes (duration NR)	NR
Pandey et al. [23] (2018)	87.5		45	Male	III	Palatal MGFP	R	CPD with buccal guide flange	R	Not done	Yes	Yes	NR	NR
Gupta et al. [24] (2020)	87.5		35	Male	III	Palatal MGFP	R	NR	NR	Yes (PMMC)	NR	NR	3 months	NR
Mahamune et al. [25] (2015)	87.5		64	Female	III	Palatal MGFP	R	NR	NR	NR	Yes	NR	1 year	NR
Adaki et al. [26] (2015)	87.5		55	Male	III	Speech prosthesis with guide flange	R	NR	NR	NR	NR	Yes	3 weeks	NR
Jamayet et al. [27] (2015)		100	28	Male	IV	CPD with MRP	R	CPD with guide flange	R	NR	NR	NR	2 months	None
Singh et al. [28] (2015)		100	76	Male	II	MRP followed by CPD	R	CPD with guide flange	R	Not done	NR	NR	2 years	None
Choudhary et al. [29] (2018)		87.5	32	Female	II	MRP	R	CPD with guide flange	R	Not done	NR	NR	1 year	None
Bandodkar et al. [30] (2021)		87.5	42	Male	III	NA	NA	MGFP	R	Not done	Yes	NR	1 year	NR
Rathee et al. [31] (2015)		87.5	60	Male	III	NA	NA	MGFP	R	Not done	NR	Yes	Yes (duration NR)	NR
Madan et al. [32] (2018)		87.5	35	Male	II	NA	NA	MGFP	R	Yes (soft tissue)	Yes	NR	1 month	None
Tandan et al. [33] (2015)		62.5	53	Male	III	NR	NR	MGFP	R	NR	NR	NR	3 weeks	NR
Mohapatra et al. [34] (2020)		87.5	45	Male	III	Stabilization plate	R	MGFP	R	Yes (PMMC)	NR	Yes	NR	NR
Bahri et al. [35] (2021)		87.5	53	Male	II	NR	NR	MGFP with MMF screws	R	NR	Yes	Yes	3 weeks	None
Hazari and Gaikwad [36] (2014)		70.5	36	Female	III	NR	NR	CPD with guide flange	R	NR	NR	NR	NR	NR
Jain [37] (2018)		100	45	Male	IV	NA	NA	RPD with MGFP	R	Yes (FG)	Yes	NR	1 year	None
Suganna et al. [38] (2019)		87.5	36	Male	II	NA	NA	MGFP followed by CPD	R	Yes (Ti plate)	NR	NR	Yes (duration NR)	Yes and corrected
Chhuchhar and Gandhewar [39] (2013)		87.5	71	Male	III	NA	NA	Modified MGFP	R	NR	NR	Yes	1 year 6 months	NR
Adaki et al. [40] (2016)		70.5	52	Male	III	MRP	NR	NR	NR	Yes (PMMC)	NR	Yes	2 weeks	NR
Singh et al. [41] (2015)		70.5	65	Male	II	CD with MRP	R	CD	R	Yes (mucocutaneous flap)	NR	Yes	Yes (duration NR)	Yes and corrected
Prasad et al. [42] (2013)		70.5	45	Male	III	Obturator with MRP	R	NR	NR	NR	NR	NR	Yes (duration NR)	Yes and corrected
Bhochhibhoya et al. [43] (2016)	100		45	Female	III	NA	NA	Hollow CPD with polystyrene	R	Yes (Ti plate)	NR	NR	every 6 months	Yes and corrected
Shah et al. [44] (2017)	100		16	Female	IV	NA	NA	Modified swing lock CPD	R	Yes (IG)	NR	NR	6 months	None
Muralidhar et al. [45] (2011)	70.5		20	Female	IV	NA	NA	CPD with buccal extension	R	Yes (bone graft)	NR	NR	3 months	Yes and corrected
Daniel et al. [46] (2019)	87.5		30	Female	IV	NA	NA	CPD	R	Yes (rib graft)	Yes	NR	Yes (duration NR)	NR
Gowda et al. [47] (2020)	100		69	Female	III	NA	NA	CPD with salivary reservoir	R	Yes (PMMC)	Yes	Yes	NR	NR
Mundhe et al. [48] (2014)	100		58	Male	I	NA	NA	CPD with attachment	R	NR	NR	Not done	1 year	None
Prasad et al. [49] (2016)	87.5		74	Female	I	NR	NR	CD with snap-fit button	R	NR	NR	NR	1 week	None
Belkhode et al. [50] (2019)	100		56	Male	III	RPD	R	Overlay CD	R	NR	Yes	Yes	24 hours	Yes and corrected
Aruna and Thulasingam [51] (2013)	87.5		40	Female	IV	NA	NA	MGFP, overlay denture	R	Yes (PMMC)	NR	Yes	Yes (duration NR)	None
Gandhimathi et al. [52] (2015)	62.5		25	Female	III	Flexible denture-ARC	R	Flexible denture, ARC	R	NR	NR	NR	3 months	NR
Rathee et al. [53] (2014)	75		55	Male	II	CPD with hollow bulb obturator and guide flange	R	RPD	R	NR	NR	Yes	NR	NR
Kumar et al. [54] (2013)	87.5		45	Male	II	Obturator	R	MGFP	R	NR	NR	NR	4 weeks	NR
Aggarwal et al. [55] (2014)	87.5		49	Male	III	Palatal MGFP	R	None	R	Yes (PMMC)	NR	NR	3 weeks	NR

**Table 4:** Summary of the data collected from case series NA: not applicable; NR: not reported; R: removable; F: fixed; CCC: Cantor-Curtis classification; JBI: Joanna Briggs Institute; MRP: maxillary ramp prosthesis; MGFP: mandibular guide flange prosthesis; RPD: removable partial denture; RT: radiotherapy; CPD: cast partial denture.

Authors	JBI (%)	Age	Sex	CCC	Maxilla		Mandible		Mandibular Reconstruction	Surgical Scarring	RT	Recall Visit	Adverse Effect
					Name of Prosthesis	Type of Prosthesis	Name of Prosthesis	Type of Prosthesis					
Hazra et al. [56] (2021)	70	43	Male	II	NA	NA	MGFP	R	NR	NR	NR	2 months	None
		47	Male	II	Guide flange with MRP	R	NR	NR	NR	NR	NR	3 months	None
Kar et al. [57] (2015)	60	27	Male	II	NA	NA	CPD with MGFP	R	NR	NR	NR	1 year	None
Lingeswar et al. [58] (2017)	70	36	Male	II	NA	NA	MGFP with clasp	R	Yes (myocutaneous flap)	Yes	NR	1 month	None
		49	Male	III	MRP with functionally generated occlusion	R	NR	NR	Yes (myocutaneous flap)	NR	Yes	1 month	NR
Jain et al. [59] (2012)	60	25	Female	II	MRP with palatally positioned teeth	R	RPD	R	NR	NR	NR	NR	NR
Gupta et al. [60] (2012)	70	55	Female	III	Guide flange with MRP	R	NR	NR	NR	Yes	Yes	6 months	Yes and corrected
		27	Male	II	Guide flange with MRP	R	RPD	R	NR	Yes	Yes	NR	NR

Prosthetic Rehabilitation

Implant-supported prosthesis (ISP) was adopted in four studies [7-10]. Thirty-eight cases used guide flange (palatal/mandibular (MGFP)/maxillary ramp prosthesis (MRP)) for the correction of mandibular deviation, whereas twin occlusion prosthesis was delivered in 13 individuals. Interim (MGFP/MRP) followed by definitive prosthesis like cast partial denture (CPD; n=8), CPD with attachment [21] and overlay denture [51] was noted. In two cases, implant-supported overdenture was the treatment of choice [8-10].

Post-prosthetic Follow-Up

Post-prosthetic recall visits were reported in 48 cases with duration ranging between 48 hours to four years (n=9; < 1 month, n=27; 1 month to 1 year, n=4 ; >1 year). Recall visit without duration was noted in eight cases, while follow-up was not reported among 10 cases.

Discussion

Prosthodontic rehabilitation of hemimandibulectomy defects is a challenging task including multiple procedures with an interdisciplinary approach towards restoring function and patient satisfaction.

Prosthetic Alternatives According to Nature and Extent of the Defect

Prosthetic rehabilitation post-resection with radiotherapy poses challenges in implant placement due to the risk of radiation-induced osteoradionecrosis at the bone level. Dental implant placement within 12 months following radiotherapy corresponds with a 34% increased risk of failure and thus recommends placement after one year of irradiation. Radiation dose greater than 5,000 cGy increases the implant failure rate to 33% [9]. A case of class I defect exhibiting marginal mandibulectomy was successfully rehabilitated using a single-piece smooth surface cortically anchored implant-supported fixed partial denture placed in native bone. These implants are the preferred choice in post-radiotherapy cases, as they do not require active biologic osseointegration (immediate loading is possible), transmit occlusal forces at the cortical bone, reduce the risk of infection, devoid of micro gap junctions resulting in the least plaque accumulation causing peri-implantitis and no abutment screw loosening/fracture as compared to two-piece implants [7]. Removable CPD or CD with extracoronal semi-precision attachments is a cost-effective and less invasive treatment alternative where dental implants are not feasible [48,49].

Segmental mandibulectomy distal to the canine (CC class II) devoid of hard tissue reconstruction leaves the patient with no option of chewing due to mandibular deviation towards the resected side exhibiting rotation and angular path of jaw closure. This is aggravated in edentate arches due to the generation of unilateral occlusal forces during mastication resulting in dislodgement of maxillary denture; therefore, implant-supported overdenture adjunct with MRP has overcome these problems and has proved to be a boon in completely edentate patients. The abnormal jaw relations along with the angular path of closure favoured the use of monoplane teeth adjunct with neutrocentric concept are advisable to achieve a non-restrictive occlusion. The maxillary ramp stabilizes the prosthesis, limits the mandibular deviation and provides a broad occlusal table for ease in mastication [8,9]. If implants are not practical, removable MGFP/MRP are advisable where the mandible can be manipulated to correct the deviation followed by definitive prosthesis [20,21,28,29,32,34,37,41,53-60]. In individuals where mandibular deviation correction was not possible manually as mostly seen following radiotherapy and scar formation, a twin occlusion (palatal row for occlusion and buccal row for cheek support) has proved to be beneficial in achieving mastication and aesthetics [11,14,15,17,19].

A class III segmental resection extending to the midline elicits increased mandibular deviation with marked facial disfigurement, decreased masticatory function, diminished speech and altered occlusion with condylar rotation resulting in anterior open bite thereby complicating the treatment prognosis. Early initiation of post-resection jaw exercises helps to loosen the scar contracture and improve the maxillomandibular relationship [2]. Intermaxillary fixation may also minimise deviation but complicates feeding. Acrylic MGFP is a cost-effective alternative with the added advantage of periodic adjustment over metal guide flange [24-26,30,31,33,34,36,39,40,42,55,57]. The acrylic flange during sequential adjustment becomes thin and weak; hence, reinforcing with ‘W’-shaped wrought wire is an innovative approach to overcome this drawback [23]. The twin occlusion prosthesis as given in class II situation is indicated in class II defects [16,18]. In completely edentate individuals, the definitive treatment of choice is similar to class II defect. Flexible dentures (Valplast) are indicated in reduced mouth opening with mandibular deviation situations which facilitates easy insertion and removal. It comprised monoplane occlusion (minimises stress and improves stability) along with acetal resin clasp to enhance retention and aesthetics [52].

Class IV defect involves the resection of the lateral aspect of the mandible augmented to maintain pseudo articulation of bone and soft tissue in the region of the ascending ramus. It presents with facial asymmetry, mandibular deviation and improper occlusion due to the depressor muscle action of the normal side. Various treatment modalities have been proposed to reduce post-surgical mandibular deviation (e.g. mandibular guidance therapy, intermaxillary fixation, resection guidance restorations). A similar challenging situation with limited interarch distance causing occlusal interference by the buccal flange of MGFP on the non-resected side has been resolved by using an MRP in relation to the non-defect side and MGFP on the defect side to establish bilateral guidance. This unique combination of prosthesis reduces the deviation and retrains the individual to achieve proper occlusion by neuromuscular reprogramming activity [27].

Authors have suggested immediate mandibular reconstruction post-resection with vascularised free flaps to prevent implant placement complications post-radiotherapy [9] and improve both facial symmetry and masticatory efficiency. Implant prosthesis is the treatment of choice for a reconstructed mandible, but an extensive period of more than one year is required for the healing of osseous graft and osseointegration of implants (in irradiation). During this initial healing phase, early prosthodontic intervention by MGFP and maxillary stabilisation prosthesis serve the purpose of reducing the mandibular deviation, preventing extrusion of the maxillary teeth and improving the masticatory efficiency. In cases where implants are not feasible, an interim MGFP/MRP followed by CPD is an effective, economic alternative [45,46]. A modified swing lock CPD with the flexible arc of the acetal labial bar has been used by authors which supersede the conventional complex design with enhanced aesthetics along with retention and stability [43]. Mandibular deviation and anterior open bite in a complex class IV defect (along with radical neck dissection and base of the tongue) can be corrected with MGFP subsequently followed by a definitive prosthesis. In some patients, the correction of mandibular rotation with a guidance appliance cannot be accomplished adequately; therefore, to compensate for the residual open bite, an overlay RPD provides optimum occlusion bilaterally improving the patient’s form and function [51].

The present systematic review had limitations as it was restricted only to Medline by means of PubMed and Google Scholar; so, the literature published on other databases and languages apart from English may have been omitted despite meeting all our inclusion criteria. Randomised controlled trials (RCT) were scarce in our search on the particular topic; therefore, the next level of evidence (i.e. case reports and series) was included; therefore, authors are urged to perform extensive RCTs on similar topics. Post-prosthesis observation duration was a deficit in many studies, while few reported short-term (less than one month) follow-up; therefore, future studies with long-term follow-up data are recommended for assessing the prosthesis longevity. Several data were lacking from the reviewed literature including CC classification, reconstruction type and scarring which decreases the article quality limiting us to deduce a strong correlation among the type of prosthesis to be selected for a particular situation.

## Conclusions

This study suggests that the first line of treatment following surgical resection includes hard tissue graft reconstruction along with interim guidance and definitive implant prosthesis (after one year in case of irradiation). In cases of CC classes II, III, IV and V defects associated with mandibular deviation where occlusion can be attained manually, MGFP/MRP would be the preferred treatment modality followed by a definitive prosthesis. In mandibular-deviated cases where occlusion is not achievable manually, a twin occlusion prosthesis is recommended.

In spite of certain limitations, this review highlights numerous prosthetic approaches according to the extent of hemimandibular defect and type of reconstruction in a snapshot. Adequate patient adaptation with minor complications in few which when corrected revealed favourable results. This study will guide the clinicians during treatment planning of hemimandibulectomy patients.

## Appendices

JBI critical appraisal checklist for case reports

Reviewer   ____________________________________ Date____________________________

Author ____________________________________ Year_________ Record Number_________

                                                                                                                             Yes    No      Unclear     NA

1. Were the patient’s demographic characteristics clearly described?              □       □             □           □

2. Was the patient’s history clearly described and presented as a timeline?   □        □             □          □

3. Was the current clinical condition of the patient on presentation

     clearly described?                                                                                             □        □             □          □

4. Were diagnostic tests or assessment methods and the results

     clearly described?                                                                                             □         □            □          □

5. Was the intervention(s) or treatment procedure(s) clearly described?          □         □             □         □

6. Was the post-intervention clinical condition clearly described?                    □         □             □         □

7. Were the adverse events (harms) or unanticipated events identified

     and described?                                                                                                 □         □             □          □

8. Does the case report provide takeaway lessons?                                          □         □             □          □

Overall appraisal:                 Include   □         Exclude  □         Seek further info  □

Comments (Including reason for exclusion)______________________________________

JBI critical appraisal checklist for case series

Reviewer   ____________________________________ Date____________________________

Author _____________________________________ Year_________ Record Number________

                                                                                                                   Yes      No      Unclear     NA

1. Were there clear criteria for inclusion in the case series?                   □         □             □            □

2. Was the condition measured in a standard, reliable way 

     for all participants included in the case series?                                  □         □             □             □

3. Were valid methods used for the identification of the

     condition for all participants included in the case series?                 □         □             □            □

4. Did the case series have consecutive inclusion of participants?       □         □             □            □

5. Did the case series have a complete inclusion of participants?         □         □             □           □

6. Was there clear reporting of the demographics of the participants

     in the study?                                                                                           □         □             □           □

7. Was there clear reporting of clinical information of the

     participants?                                                                                           □          □             □            □

8. Were the outcomes or follow-up results of cases clearly reported?   □         □              □            □

9. Was there clear reporting of the presenting site(s)/clinic(s)

     demographic information?                                                                     □         □              □           □

10. Was statistical analysis appropriate?                                                    □         □              □           □

Overall appraisal:                 Include   □         Exclude  □         Seek further info  □

Comments (Including reason for exclusion)_______________________________________
